# Circular RNA circCSPP1 promotes the occurrence and development of colon cancer by sponging miR-431 and regulating ROCK1 and ZEB1

**DOI:** 10.1186/s12967-022-03240-x

**Published:** 2022-01-31

**Authors:** Jin Wang, Lei Zhou, Bingxin Chen, Zhuwen Yu, Jianglei Zhang, Zhe Zhang, Chenrui Hu, Yanjin Bai, Xiaokang Ruan, Shengjia Wang, Jun Ouyang, Airong Wu, Xin Zhao

**Affiliations:** 1 Department of General Surgery, The First Affiliated Hospital of Soochow University, Suzhou, China; 2 Department of General Surgery, Suzhou Dushu Lake Hospital (Dushu Lake Hospital Affiliated to Soochow University), Suzhou, China; 3 Jiangsu Institute of Clinical Immunology, The First Affiliated Hospital of Soochow University, Suzhou, China; 4 Jiangsu Key Laboratory of Clinical Immunology, Soochow University, Suzhou, China; 5 Jiangsu Key Laboratory of Gastrointestinal Tumor Immunology, Suzhou, China; 6 Department of Gastroenterology, Suzhou Hospital of Integrated Traditional Chinese and Western Medicine, Suzhou, China; 7 Department of Gastroenterology, The First Affiliated Hospital of Soochow University, Suzhou, China; 8 Department of Urinary Surgery, The First Affiliated Hospital of Soochow University, Suzhou, China

**Keywords:** circCSPP1, miR-431, Rho associated coiled-coil containing protein kinase 1, Zinc finger E-box binding homeobox 1, Colon cancer, Cell cycle, Epithelial-to-mesenchymal transition

## Abstract

**Background:**

Colon cancer is a common malignant tumor of the digestive tract, and its incidence is ranked third among gastrointestinal tumors. The present study aims to investigate the role of a novel circular RNA (circCSPP1) in colon cancer and its underlying molecular mechanisms.

**Methods:**

Bioinformatics analysis and reverse transcription-quantitative PCR were used to detect the expression levels of circCSPP1 in colon cancer tissues and cell lines. The effects of circCSPP1 on the behavior of colon cancer cells were investigated using CCK-8, transwell and clonogenic assays. Bioinformatics analysis along with luciferase, fluorescence in situ hybridization and RNA pull-down assays were used to reveal the interaction between circCSPP1, microRNA (miR)-431, Rho associated coiled-coil containing protein kinase 1 (ROCK1) and zinc finger E-box binding homeobox 1 (ZEB1).

**Results:**

It was found that circCSPP1 expression was significantly upregulated in colon cancer tissues and cell lines. Overexpression of circCSPP1 significantly promoted the proliferation, migration and invasion of colon cancer cells, whereas silencing of circCSPP1 exerted opposite effects. Mechanistically, circCSPP1 was found to bind with miR-431. In addition, ROCK1 and ZEB1 were identified as the target genes of miR-431. Rescue experiments further confirmed the interaction between circCSPP1, miR-431, ROCK1 and ZEB1. Moreover, circCSPP1 promoted the expression level of ROCK1, cyclin D1, cyclin-dependent kinase 4, ZEB1 and Snail, and lowered the E-cadherin expression level.

**Conclusion:**

Taken together, the findings of the present study indicated that circCSPP1 may function as a competing endogenous RNA in the progression of colon cancer by regulating the miR-431/ROCK1 and miR-431/ZEB1 signaling axes.

**Supplementary Information:**

The online version contains supplementary material available at 10.1186/s12967-022-03240-x.

## Background

Colon cancer is a common malignant tumor of the digestive tract, and its incidence is ranked third among gastrointestinal tumors [1]. Over the past few decades, the rapid development of molecular biology has enriched the theory of colorectal cancer carcinogenesis [1–4]. In addition, immense progress has been made in diagnostic and treatment strategies for colorectal cancer; the 5-year survival rate of patients with localized disease is 90.1% [5]. However, following the metastasis of colorectal cancer to adjacent organs or lymph nodes, the 5-year survival rate of patients decreases to 69.2%. Of note, only 39% of patients with colorectal cancer are diagnosed at the localized stage of the disease, prior to metastasis [6, 7]. Therefore, further in-depth investigations of the pathogenesis of colorectal cancer, as well as the identification of more effective early diagnosis and treatment strategies are essential for colorectal cancer.

MicroRNAs (miRNAs/miRs) are small endogenous single-stranded RNA molecules composed of ~ 20 nucleotides, which act mostly on the 3′UTR of target mRNAs and either degrade or inhibit multiple transcripts [8, 9]. Previous studies have demonstrated that miRNAs play a critical regulatory role in the initiation and progression of human cancers [10, 11].

Circular RNAs (circRNAs) are newly discovered non-coding RNAs with a covalently closed ring structure, which are widely found in a variety of cells [12–15]. They are produced by the reverse splicing of precursor mRNAs and characterized by a stable structure, a conserved sequence and tissue specificity. Recent studies have indicated that circRNAs can act as miRNAs sponges to inhibit the activity of targeted miRNAs [16, 17]. In addition, circRNAs can regulate gene transcription by binding with RNA binding proteins, or can be translated to produce proteins [18]. Thus, circRNAs play a vital role during the progression of tumors, and may provide a novel direction for tumor diagnosis and therapy [19, 20].

In the present study, the commonly differentially expressed circRNAs between colon cancer tissues and adjacent normal tissues in two public datasets were screened out to identify novel molecular targets for colon cancer treatment. It was found that circCSPP1 was significantly upregulated in cancer tissues. In addition, the role of circCSPP1 in colon cancer was examined in vitro and in vivo.

## Materials and methods

### Specimen collection

Cancer tissues and adjacent normal tissues were collected from 25 patients (14 male, 11 female), who diagnosed with colon cancer at the First Affiliated Hospital of Soochow University (Suzhou, China) (August, 2020 to July, 2021). These patients received no treatment before and age of them was range from 37 to 72-year-old. The tissues were stored in liquid nitrogen immediately after resection. The present study was approved by the Ethics Committee of the First Affiliated Hospital of Soochow University (No. FAHSU20200719) and written informed consent was obtained from each patient.

### Gene expression omnibus (GEO) data analysis

The present study analyzed the GSE121895 and GSE126094 datasets from the GEO database. The expression levels in each group were normalized. The threshold value of differentially expressed genes was set at two of different multiples and p < 0.05.

### Cell culture and transfection

The human colonic epithelial cell line (HFC) was purchased from ScienCell Research Laboratories, Inc. Colon cancer cell lines, including SW620, SW480, LOVO, HCT116 and DLD-1 cells were obtained from the American Type Culture Collection (ATCC). THP-1 cells were also obtained from ATCC. The cells were maintained in DMEM (Thermo Fisher Scientific, Inc.) containing 10% FBS supplemented with 100 U/ml penicillin and 100 g/ml streptomycin (Beyotime Institute of Biotechnology) at 37 °C. When the cell density (SW620, LOVO) reached 50–70%, the cells were transfected with miR-431 mimics (20 nM), mimics control, circCSPP1 pcDNA3.1 overexpression plasmid (1 μg/μl) or circCSPP1 pLVX-IRES-Puro silencing plasmid (shRNA1 and shRNA2; 1 μg/μl) for 24 h using Lipofectamine^®^ 3000 (Thermo Fisher Scientific, Inc.) according to the manufacturer’s instructions. The miR-431 mimics, miR-control, circCSPP1 pcDNA3.1 overexpression plasmid, circCSPP1 pLVX-IRES-Puro silencing plasmids; Rho associated coiled-coil containing protein kinase 1 (ROCK1) and ZEB1 pLVX-IRES-Puro silencing plasmids were obtained from Shanghai Genepharma Co., Ltd. Phorbol-12-myristate-13-acetate (PMA), IL-4 and IL-13 were purchased from Sigma-Aldrich; Merck KGaA. The information of oligonucleotide was provided in Table 1.

**Table 1:** The information of oligonucleotide sequences

Gene	Sequence (5′–3′)
ROCK1 shRNA	sense: CACCGCATTTGGAGAAGTTCAATTGCGAACAATTGAACTTCTCCAAATGC antisense: AAAAGCATTTGGAGAAGTTCAATTGTTCGCAATTGAACTTCTCCAAATG
ZEB1 shRNA	sense: CACCGAGAGAGAGAGTTTGACAAGGCGAACCTTGTCAAACTCTCTCTCTC
	antisense: AAAAGAGAGAGAGAGTTTGACAAGGTTCGCCTTGTCAAACTCTCTCTCTC
circCSPP1 shRNA1	sense: CACCGCTCCAGACAATGAAACATCCCGAAGGATGTTTCATTGTCTGGAGC antisense: AAAAGCTCCAGACAATGAAACATCCTTCGGGATGTTTCATTGTCTGGAGC
circCSPP1 shRNA2	sense: CACCGCTAATCAAGATACCTGTAGTCGAAACTACAGGTATCTTGATTAGC antisense: AAAAGCTAATCAAGATACCTGTAGTTTCGACTACAGGTATCTTGATTAGC
shRNA control	sense: GATCCGTTCTCCGAACGTGTCACGTTTCAAGAGAACGTGACACGTTCGGAGAACTTTTTTG antisense: AATTCAAAAAAGTTCTCCGAACGTGTCACGTTCTCTTGAAACGTGACACGTTCGGAGAACG
miR-431 mimic	UGUCUUGCAGGCCGUCAUGCA
mimic control	UUCUCCGAACGUGUCACGUTT
miR-431 inhibitor	UGCAUGACGGCCUGCAAGACA
inhibitor control	UUCUCCGAACGUGUCACGUTT
miR-324-5p mimic	CGCAUCCCCUAGGGCAUUGGUG
miR-375 mimic	UUUGUUCGUUCGGCUCGCGUGA
miR-486-3p mimic	CGGGGCAGCUCAGUACAGGAU

### Reverse transcription-quantitative PCR (RT-qPCR)

TRIzol® reagent (Thermo Fisher Scientific, Inc.) was used to extract the RNA according to the manufacturer’s protocol. The RNA was reverse transcribed into cDNA using a reverse transcription kit (Takara Bio, Inc.). The expression levels of miR-431, ROCK1 and zinc finger E-box binding homeobox 1 (ZEB1) were detected using a fluorescence quantitative PCR kit (Nanjing Jiancheng Bioengineering Inc.) in a BD FACSVerse™ (BD Biosciences). U6 and GAPDH were used as internal controls for miR-431 and mRNAs, respectively. Real-Time qPCRs were used three times: 2 min at 94 °C, followed by 35 cycles (94 °C for 30 s and 55 °C for 45 s). The primers used were as follows: RT primer for miR-431, 5′-GTCGTATCCAGTGCAGGGTCCGAGGTGCACTGGATACGACACGUACU-3′; miR-431 forward, 5′-TGCGGUGUCUUGCAGGCCGUCAG-3′ and reverse, 5′-CCAGTGCAGGGTCCGAGGT-3′; U6 forward, 5′-CTCGCTTCGGCAGCACA-3′ and reverse, 5′-AACGCTTCACGAATTTGCGT-3′; ROCK1 forward, 5′-AACATGCTGCTGGATAAATCTGG-3′ and reverse, 5′-TGTATCACATCGTACCATGCCT-3′; ZEB1 forward, 5′-TTCTCACACTCTGGGTCTTATTCTC-3′ and reverse, 5′-CTTTTTCACTGTCTTCATCCTCTTC-3′; arginase-1 forward, 5′-AGACCACAGTTTGGCAATTGG-3′ and reverse, 5′-AGGAGAATCCTGGCACATCG-3′; IL-10 forward, 5′-AACCTGCCTAACATGCTTCG-3′ and reverse, 5′-GAGTTCACATGCGCCTTGAT-3′; circCSPP1 forward, 5′-CCATCCCATCAGTTCATCCT-3′ and reverse, 5′-CCCTGCAAAAGGACTACAGG-3′; SMAD4 forward, 5′-GCTGCTGGAATTGGTGTTGA-3′ and reverse, 5′-CTTCGTCTAGGAGCTGGAGG-3′; DAAM1 forward, 5′-TTCATTCATCTTTTGCTGTTTCCGA-3′ and reverse, 5′-TTTTCTTCCTGGTCCTTTTTCTTGC-3′; CDK14 forward, 5′-GCACAGAGACCTGAAACCACAG-3′ and reverse, 5′-AAAGATGCAACCTACTCCCCAC-3′; GAPDH forward, 5′-TCAAGAAGGTGGTGAAGCAGG-3′ and reverse, 5′-TCAAAGGTGGAGGAGTGGGT-3′. The data were quantified by using 2^−ΔΔt^ method [21]. All these experiments were performed in triplicate.

### Cell counting kit-8 (CCK-8) assay

The SW620 or LOVO cells (3 × 10^5^) were seeded in 96-well plates and cultured for 0, 24, 48 and 72 h. At each time point, the cells were incubated with 10 μl CCK-8 solution (Beyotime) at 37 °C for 4 h. The optical density was then measured at 450 nm as previously described [22]. All these experiments were performed in triplicate.

### Cell colony forming assay

The cells (5 × 10^3^) were suspended in DMEM containing 10% FBS, and then seeded into the plate. After 2 weeks of incubation at 37 °C, the cells were fixed with 5 ml 4% paraformaldehyde for 15 min. The cells were then stained with Giemsa (Beyotime) for 30 min. The number of colonies was counted using a light microscope (200×; Nikon Corporation). All these experiments were performed in triplicate.

### Transwell assay

The cells (2 × 10^4^) were digested and cultured in a serum-free medium in the Transwell (BD) upper chamber with or without Matrigel (BD Biosciences). Subsequently, 600 μl complete medium (10% serum) were added to the lower chamber. After 24 h of incubation at 37 °C, the cells in the lower chamber were fixed with 4% formaldehyde for 10 min at room temperature and stained with 0.1% crystal violet solution at room temperature for 10 min (Sigma-Aldrich; Merck KGaA). Finally, the migrated or invaded cells were photographed using a light microscope (200×) [23]. All these experiments were performed in triplicate.

### Fluorescence in situ hybridization (FISH) assay

Cy3-labeled circCSPP1 and FITC-labeled miR-431 probes (Biosense Technologies) were used to observe the co-localization of circCSPP1 and miR-431 in the cells. Hybridizations were performed according to the manufacturer’s instructions provided with the fluorescence in situ hybridization kit. The cell nuclei were stained with DAPI at room temperature for 20 min. Subsequently, images were visualized using a fluorescence microscope (200×) as previously described [24].

### Luciferase assay

The luciferase assay was performed using the dual-luciferase reporting system psiCHECK (Thermo Fisher Scientific, Inc.). The wild-type (WT) or mutant-type (mut) sequences of circCSPP1, ROCK1 and ZEB1 were cloned into the psiCHECK2 plasmid. 293 T cells (ATCC, 2 × 10^4^ cells/well) were cultured overnight in 24-well plates. The cells were transfected with the WT or mut reporter vector along with miR-431 mimics (10 nM) or mimics control (10 nM) using Lipofectamine^®^ 3000 (Thermo Fisher Scientific, Inc.). Finally, the luciferase activity of cells was detected with a Dual-Luciferase Detection kit (Promega Corporation) after 48 h of transfection. The data were quantified by normalizing to Renilla luciferase activity.

### RNA pull-down assay

Biotin labeled miR-431 and the control probes were synthesized by Sangon Biotech (Shanghai) Co., Ltd. Probe-coated beads were generated by co-incubation with streptavidin-coated beads (Thermo Fisher Scientific, Inc.) at 25 °C for 2 h. The SW620 and LOVO cells were collected, lysed and incubated with miR-431 probes overnight at 4 °C. Thereafter, the beads were eluted, and the complex was purified using TRIzol^®^ reagent (Takara Biotechnology Co., Ltd.). The levels of circCSPP1, ROCK1 and ZEB1 were then analyzed using RT-qPCR.

### RNA immunoprecipitation (RIP) assay

RIP assay was performed using the EZ-Magna RIP RNA-Binding Protein Immunoprecipitation kit (MilliporeSigma). Briefly, magnetic beads conjugated with negative control normal IgG (cat.no. AB21-KC, 1:5000) or anti-Ago2 (cat.no. 03-110, 1:5000) antibody (MilliporeSigma) were co-incubated with the cell lysates for 4 h at room temperature. To investigate the enrichment of the binding targets, the immunoprecipitated RNAs were extracted and subjected to RT-qPCR.

### Western blot analysis

RIPA lysis buffer (Beyotime Institute of Biotechnology) was used to extract protein from the cells. The protein concentration was determined using the BCA kit (Nanjing Jiancheng Bioengineering Inc.) according to the manufacturer’s instructions. Protein (40 μg) was then separated by using 10% SDS-PAGE, and transferred onto PVDF membranes (MilliporeSigma). The membranes were blocked in 5% skimmed milk for 1 h at room temperature followed by incubation with the following primary antibodies: ROCK1 (cat. no. #4035, 1:1,000, Cell Signaling Technology, Inc.), ZEB1 (cat.no. ab181451, 1:1,000, Abcam), cyclin D1 (cat. no. ab16663, 1:1,000, Abcam), cyclin-dependent kinase (CDK)4 (cat.no. 11026-1-AP, 1:1,000, ProteinTech Group, Inc.), p-CDK4 (1:1,000, Abcam), retinoblastoma (Rb; cat. no. ab181616, 1:1,000, Abcam), p-Rb (cat.no. ab184796, 1:1,000, Abcam), Snail (cat.no. ab216347, 1:1,000, Abcam), E-cadherin (E-cad; cat. no. 20874-1-AP, 1:1,000, ProteinTech Group, Inc.) and GAPDH (1:1500, cat. no. HRP-60004, ProteinTech Group, Inc.) at 4 °C overnight. The membranes were then incubated with HRP-labeled goat anti-rabbit secondary antibody (Abcam, cat. no. ab7090; 1:5,000) at room temperature for 1 h. Thereafter, an enhanced chemiluminescence kit (Thermo Fisher Scientific, Inc.) was used to detect protein expression. All these experiments were performed in triplicate.

### Xenograft tumor model

Nude mice (n = 24, 4–6 weeks old, 20–22 g) were obtained from the Animal center of Soochow University and randomly divided into four groups (shRNA2 ctrl, circCSPP1 shRNA2, pcDNA3.1 ctrl and pcDNA3.1-circCSPP1). All mice were housed in a SPF‑grade animal room (temperature 18–22 °C; humidity 40–60%; light/dark cycle 12/12 h each day) and had free access to food and water. The subcutaneous injection of colon cancer cells was performed after 3 days of adaptive breeding. Each mouse was subcutaneously injected with 3 × 10^6^ colon cancer cells (100 μl in PBS). Tumor size was measured every 2 days, and the major axis (a) and minor axis (b) of the tumor were measured. The tumor volume was calculated using the following formula: ab^2^/2. At the end of the experiment, the mice were sacrificed using a 40% volume/min CO_2_ and the tumors were removed, photographed and weighed. The animal experiments were approved by the Ethics Committee of the First Affiliated Hospital of Soochow University (Approval No. 20200917). The National Institutes of Health guide for the care and use of laboratory animals was strictly followed.

### Cell cycle distribution analysis

SW620 or LOVO cells (5 × 10^5^) were fixed using with 75% ethanol for 20 min on ice. Then, cells were permeabilized with 0.25% Triton X-100 and stained with PI/RNase (Sigma Aldrich). After 15 min of incubation at 4 °C, cells were analyzed using a flow cytometer (BD FACSAria III; BD Biosciences) and ModFit (version 3.0; Verity Software House, Inc.). All these experiments were performed in triplicate.

### Statistical analysis

Three independent experiments were performed in each group. Statistical analysis was performed using GraphPad Prism software (GraphPad Software, Inc.). The measurement data are expressed as the mean ± standard deviation. The unpaired Student’s t-test was used for comparisons between two groups, and One-way analysis of variance and Tukey’s post hoc tests were used for comparisons between multiple groups [25]. p < 0.05 was considered to indicate a statistically significant difference.

## Results

### CircCSPP1 is highly expressed in colon cancer

**Fig. 1:**
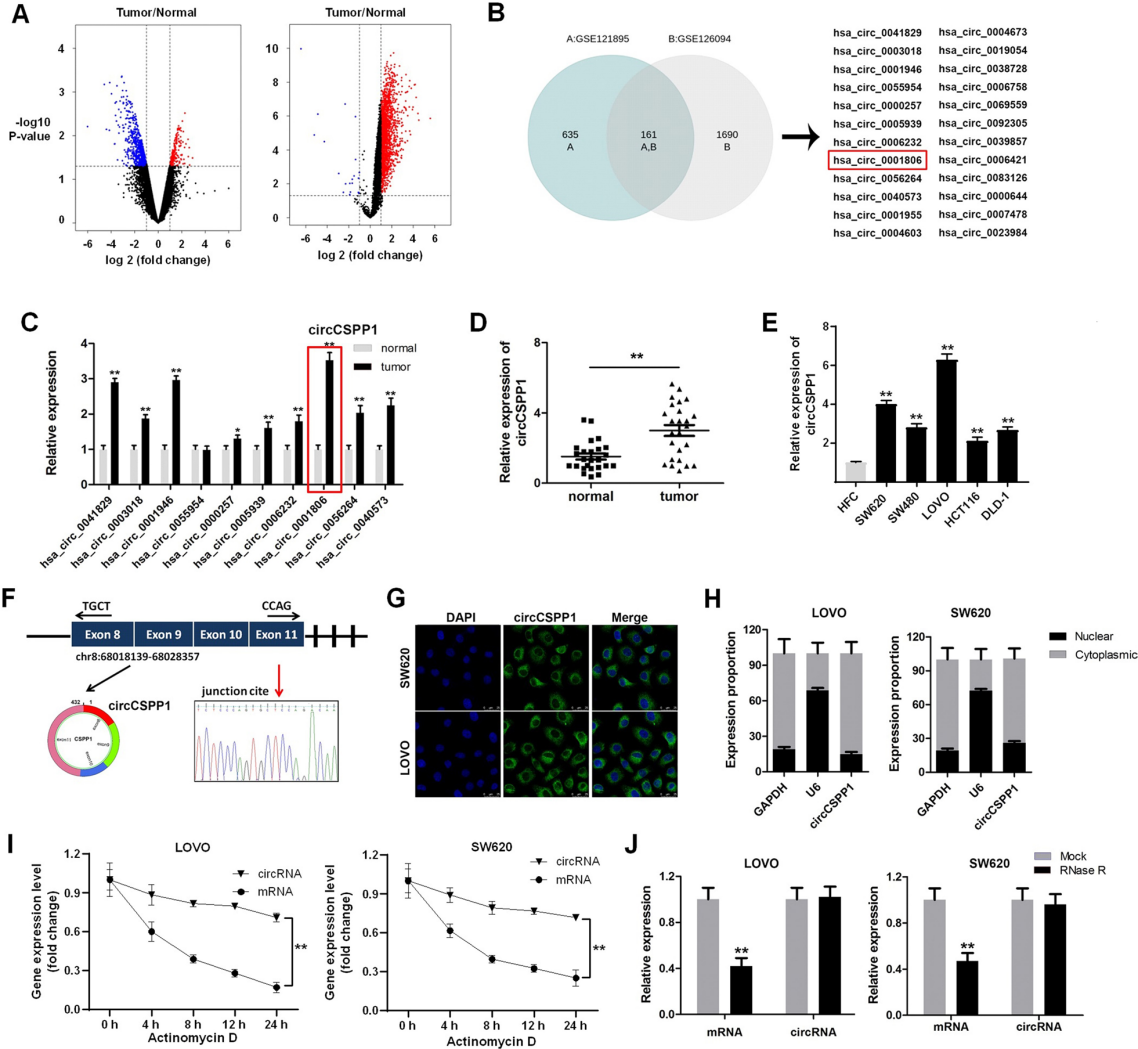
CircCSPP1 is highly expressed in colon cancer tissues. **A** GEO database data analysis of colon cancer differentially expressed circRNA. **B** Wayne analysis of commonly differentially expressed circRNA in GSE121895 and GSE126094. **C** The expression of circRNAs in tumor tissues and in adjacent normal tissue was detected with RT-qPCR. **D** The expression of circCSPP1 in tumor tissues and in adjacent normal tissue was detected with RT-qPCR. **E** The expression of circCSPP1 in cell lines was tested with RT-qPCR. **F** The circRNA circularization mechanism is formed by splicing of exons 8 and 9, and sequencing confirmed the sequence is correct. **G** The localization of circCSPP1 in colon cancer cells was detected with FISH. **H** The expression of circCSPP1 in the nucleus and cytoplasm was detected by RT-qPCR. **I** The half-live of linear and circRNA were detected by RT-qPCR after actinomycin treatment. **J** The expression of linear and circRNA linear was detected with RT-qPCR after RNase R treatment. *p < 0.05 vs normal, **p < 0.01 vs normal, HFC, mock groups; n = 3

To explore novel molecular targets for the treatment of colon cancer, the differentially expressed circRNAs between cancer and adjacent normal tissues were first analyzed using two GEO datasets (GSE121895 and GSE126094) (Fig. 1A). A total of 161 differentially expressed circRNAs were identified by intersection analysis of the two transcriptomics data (Fig. 1B). Further RT-PCR verification at the tissue level revealed that hsa_circ_0001806 (circCSPP1) was significantly upregulated in colon cancer (Fig. 1C and D). Consistently, compared with the HFC cells, the expression of circCSPP1 was found to be upregulated in colon cancer cells (Fig. 1E). In addition, circRNA circularization data indicated that circCSPP1 was spliced by exons 8–11 of the CSPP1 transcript, which was confirmed by Sanger sequencing (Fig. 1F).

Subsequently, the distribution of circCSPP1 in the cells was detected using FISH assay and RT-qPCR. The data revealed that circCSPP1 was mainly located in the cytoplasm (Fig. 1G and H). Compared with linear RNA, circCSPP1 was more resistant to actinomycin or RNase R treatment (Fig. 1I and J). These data thus indicated that circCSPP1 had higher stability and a longer half-life.

### Knockdown of circCSPP1 significantly inhibits the tumorigenesis of colon cancer

**Fig. 2:**
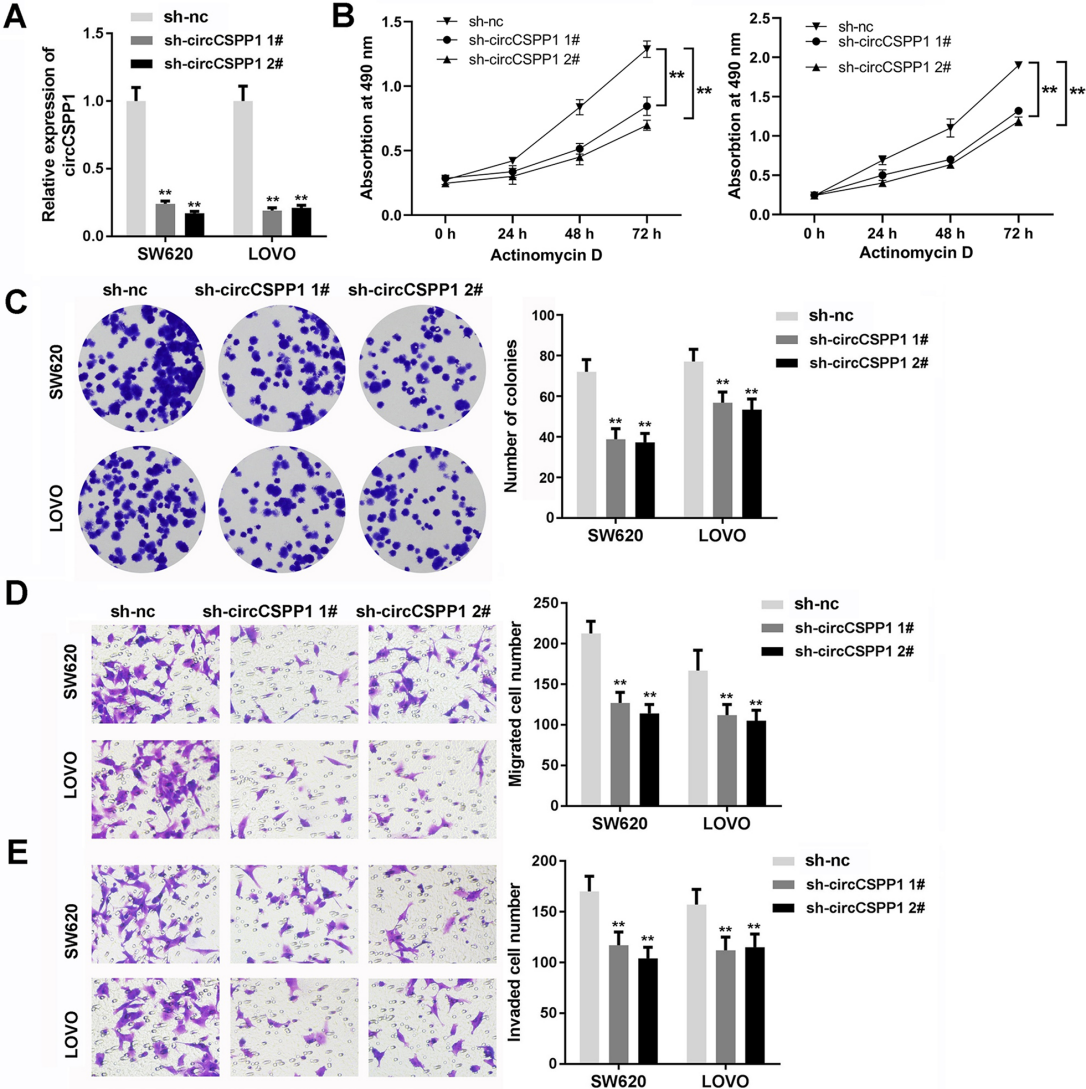
Knockdown of circCSPP1 significantly inhibited the tumorgenesis of colon cancer. Colon cancer cells were treated with circCSPP1 shRNAs or negative control (sh-nc) for 24 h. **A** The expression of circCSPP1 was detected with RT-qPCR. **B** CCK8 was used to detect the viability of colon cancer cells. **C** Cell clone formation was used to assess the proliferation of clone cancer cells. **D**, **E** The cell migration and invasion ability was detected with transwell assay. **p < 0.01 vs sh-nc; n = 3

In order to investigate the function of circCSPP1 in colon cancer, cell proliferation, invasion and migration were detected. First, circCSPP1 was knocked down in colon cancer cells using shRNA1 and shRNA2. The results of RT-qPCR revealed that both these shRNAs effectively suppressed the level of circCSPP1 in the cells (Fig. 2A). In addition, the results of the CCK-8 assay demonstrated that knockdown of circCSPP1 significantly inhibited the proliferation of colon cancer cells (Fig. 2B). Consistently, knockdown of circCSPP1 notably decreased the colony-forming ability of the cells (Fig. 2C). Moreover, transwell assay revealed that knockdown of circCSPP1 inhibited the invasive and migratory ability of colon cancer cells (Fig. 2D and E). These data thus suggested that knockdown of circCSPP1 significantly inhibited the progression of colon cancer in vitro*.*

### CircCSPP1 promotes colon cancer tumor growth and metastasis in vivo

**Fig. 3:**
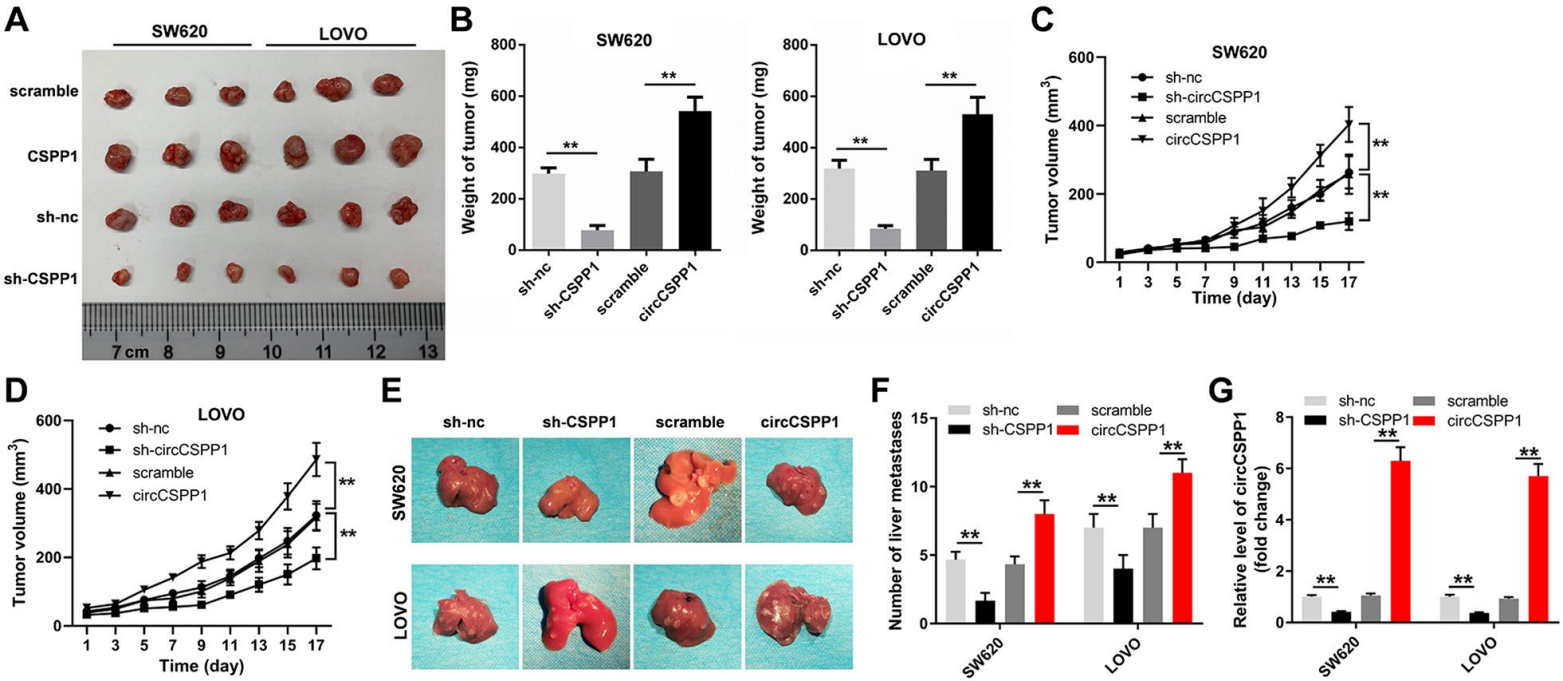
CircCSPP1 promotes colon cancer tumor growth and metastasis in vivo. **A** Tumor volume in each group was imaged in the end of animal study. **B** The tumors in mice was isolated and weighted. **C**, **D** The tumor volumes of SW620 or LOVO tumor-bearing mice were monitored every two days. **E**, **F** Liver metastasis of colon cancer in the nude mice in each group was monitored and quantified. **G** The expression of circCSPP1 in tumor tissues was detected with RT-qPCR. **p < 0.01; n = 6. PcDNA3.1 control (scramble)

With the purpose of confirming the biological function of circCSPP1 in colon cancer, an animal experiment was performed. The results of the animal experiment revealed that the overexpression of circCSPP1 significantly promoted tumor growth, whereas the knockdown of circCSPP1 inhibited tumor growth (Fig. 3A–D). Moreover, the metastasis of colon cancer in vivo was also assessed. The results showed that knockdown of circCSPP1 significantly reduced the metastasis of colon cancer, while overexpression of circCSPP1 promoted the metastasis of colon cancer (Fig. 3E and F). In addition, the expression level of circCSPP1 in tumor tissues was inhibited by circCSPP1 shRNA2 (Fig. 3G). On the whole, these indicated that circCSPP1 promoted colon cancer tumor growth and metastasis in vivo.

### CircCSPP1 sponges with miR-431 in colon cancer cells

Circinteractome database (https://circinteractome.irp.nia.nih.gov/) was used to explore the potential target of circCSPP1. A total of five miRNAs (miR-197, miR-324-5p, miR-375, miR-431 and miR-486-3p) were predicted to be the candidate targets of circCSPP1 (Additional file 1: Fig. S1A). Luciferase reporter assay was then used to screen the candidate binding miRNAs. The results revealed that the relative luciferase activities of the cells were most notably inhibited by miR-431 mimics (Additional file 1: Fig. S1A). The binding site between circCSPP1 and miR-431 is presented in Additional file 1: Fig. S1B. Subsequently, luciferase reporter assay confirmed that circCSPP1 was able to bind to miR-431 in SW620 and LOVO cells (Additional file 1: Fig. S1C). In addition, the results of FISH assay revealed the co-localization of circCSPP1 with miR-431 in the cytoplasm of the cells (Additional file 1: Fig. S1D). Moreover, RIP and RNA pull-down assays revealed that circCSPP1 could directly bind with miR-431 (Additional file 1: Fig. S1Eand F). Thus, these data suggested that circCSPP1 sponged miR-431 in colon cancer cells.

### ROCK1 is a target gene of miR-431 in colon cancer cells

Then, TargetScan (http://www.targetscan.org/vert_72/), miRDB (http://www.mirdb.org/) and miRWalk (http://zmf.umm.uni-heidelberg.de/apps/zmf/mirwalk/micrornapredictedtarget.html) were used to explore the target genes of miR-431 in colon cancer cells. The expression of several potential targets involved in cancer development was assessed. Based on these three databases, ZEB1, SMAD4, disheveled associated activator of morphogenesis 1 (DAAM1), CDK14 and ROCK1 were predicted to be the candidate targets of miR-431 (Additional file 2: Fig. S2A). ZEB1 and ROCK1 were found to be downregulated by miR-431 mimics (Additional file 2: Fig. S2A). Among these genes, ROCK1 was first selected for further analysis due to its critical role in the progression of cancer [26].

RT-qPCR results revealed that ROCK1 expression was notably upregulated in colon cancer tissues compared with adjacent normal tissues (Additional file 2: Fig. S2B). The potential complementary pairing sequence between miR-431 and the 3′-UTR of ROCK1 is presented in Additional file 2: Fig. S2C. In addition, luciferase reporter assay indicated that the luciferase activity of the cells carrying the WT ROCK1 3′-UTR was significantly reduced by miR-431 mimics (Additional file 2: Fig. S2D). Consistently, the results of RIP and RNA pull-down assays revealed the direct interaction between miR-431 and ROCK1 (Additional file 2: Fig. S2E and F). On the whole, these data confirmed that ROCK1 was a target gene of miR-431 in colon cancer cells.

### Knockdown of ROCK1 reverses the tumor-promoting effects of circCSPP1

**Fig. 4:**
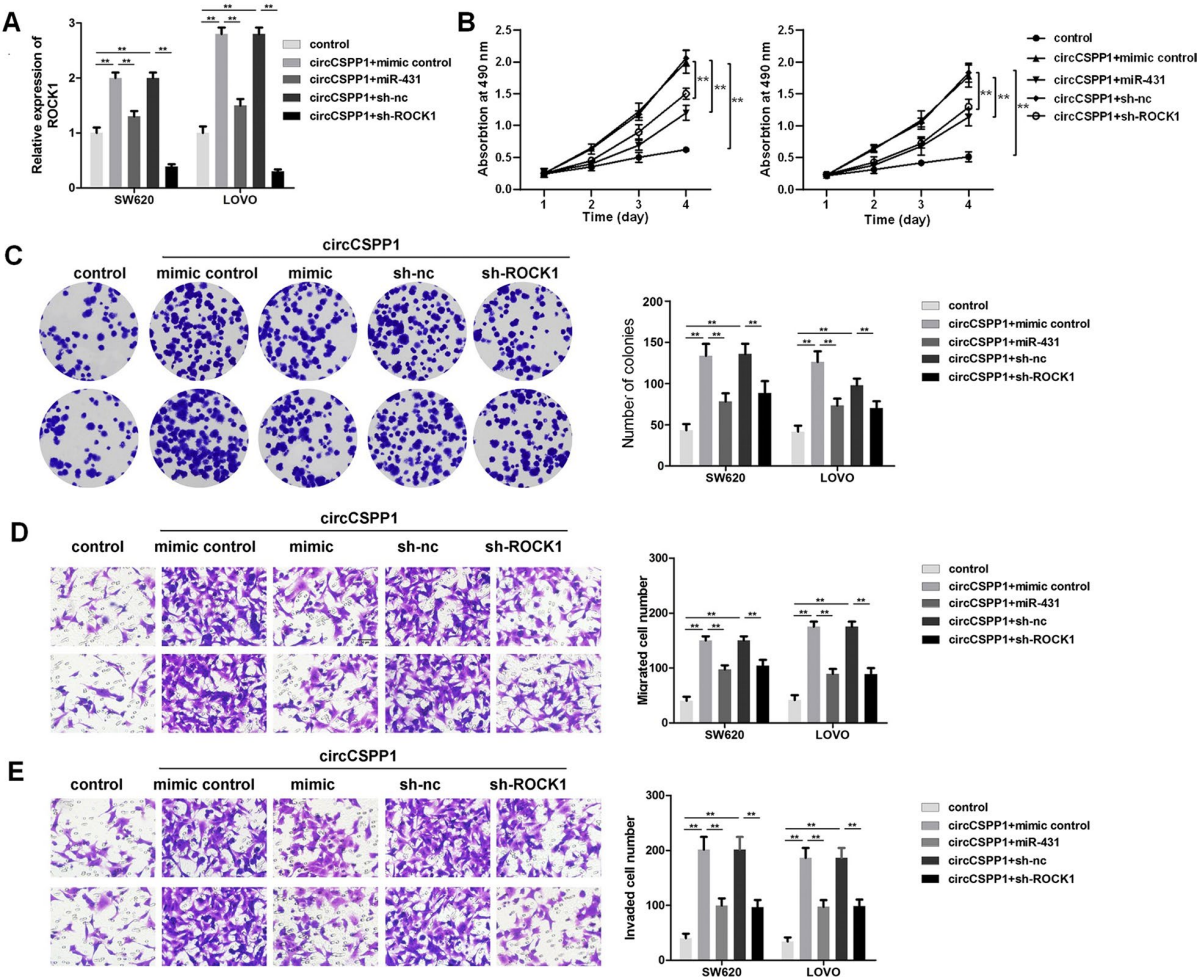
Knockdown of ROCK1 reverses the tumor-promoting effect of circCSPP1. Colon cancer cells were treated with pcDNA3.1-circCSPP1, pcDNA3.1-circCSPP1 plus miR-431 mimics or pcDNA3.1-circCSPP1 plus ROCK1 shRNA. **A** The expression of ROCK1 was detected with RT-qPCR. **B** CCK8 was used to detect the cell viability in each group. **C** Cell clone formation was used to assess the proliferation of clone cancer cells. **D**, **E** Cell migration and invasion were detected with transwell assays. **p < 0.01; n = 3

To further confirm the interaction among circCSPP1, miR-431 and ROCK1, rescue experiments were performed. The results of RT-qPCR revealed that the overexpression of circCSPP1 promoted ROCK1 expression, whereas this effect was reversed by transfection with miR-431 mimics (Fig. 4A). In addition, the results of CCK-8 and colony formation assays indicated that circCSPP1 significantly increased the proliferation of colon cancer cells, which was reversed by transfection with miR-431 mimics or ROCK1 knockdown (Fig. 4B and C). Consistently, the transwell assay results revealed that circCSPP1 notably promoted the migration and invasion of colon cancer cells, whereas these effects were reversed by miR-431 mimics or ROCK1 knockdown (Fig. 4D and E). Additionally, the effects of circCSPP1, sh-ROCK1 or sh-ZEB1 on their target genes in cells were detected with RT-qPCR, respectively (Additional file 4: Fig. S4A–C). Meanwhile, miR-431 mimics significantly increased the level of miR-431, while miR-431 inhibitor exhibited completely opposite effect (Additional file 4: Fig. S4D and F). Taken together, these findings demonstrated that the knockdown of ROCK1 reversed the tumor-promoting effects of circCSPP1.

### MiR-431 targets ZEB1 in colon cancer cells

**Fig. 5:**
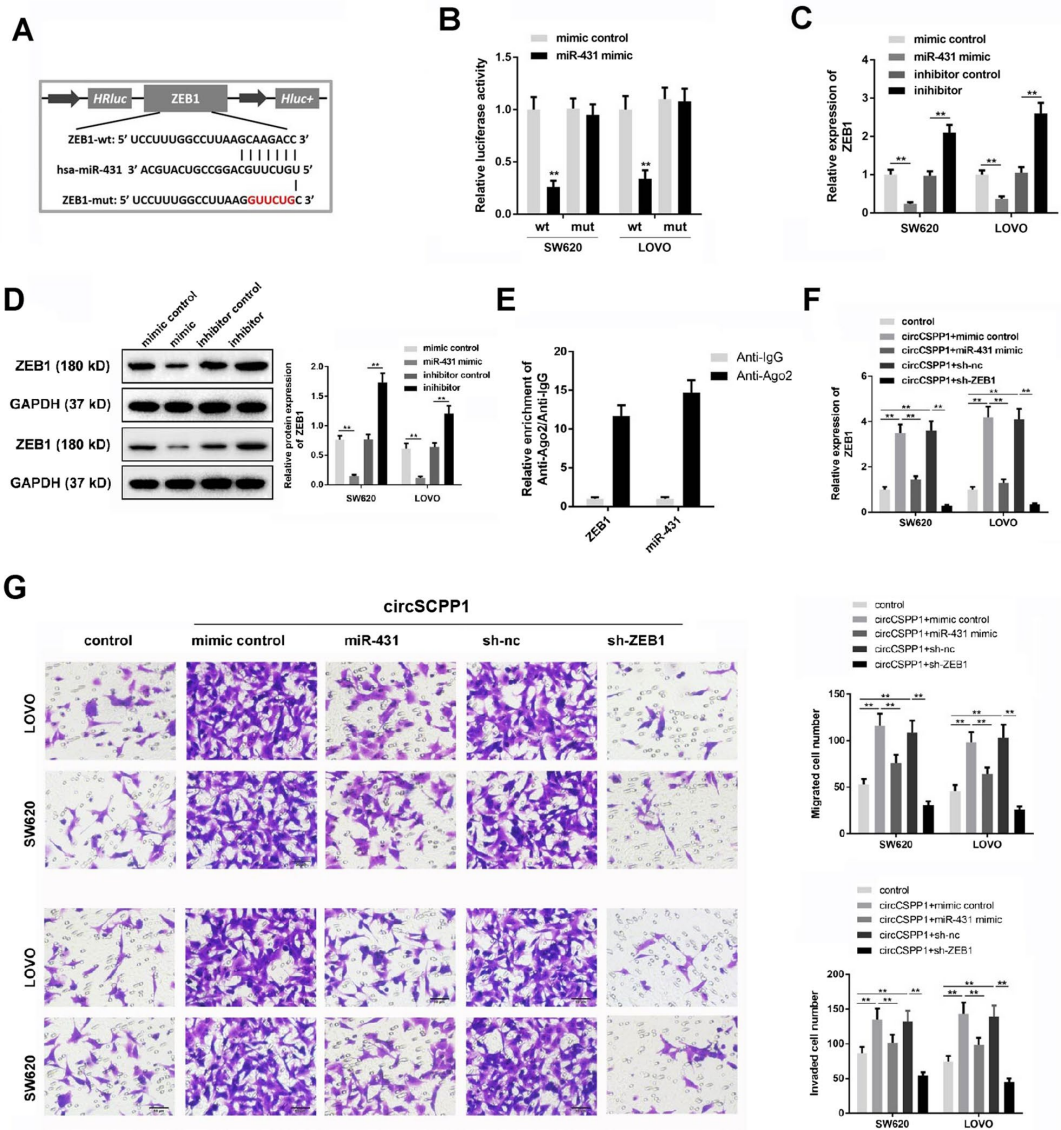
MiR-431 targets ZEB1 in colon cancer cells. **A** The binding site between miR-431 and ROCK1 was predicted. **B** Luciferase reporter experiment was performed to detect the interaction between miR-431 and ZEB1. **C** and **D** colon cancer cells were treated with miR-431 mimics or miR-431 inhibitor for 24 h, the gene and protein level of ZEB1 was detected with RT-qPCR and WB, respectively. **E** RIP assay was performed to verify the binding between ZEB1 and miR-431. Colon cancer cells were treated with pcDNA3.1-circCSPP1, pcDNA3.1-circCSPP1 plus miR-431 mimics or pcDNA3.1-circCSPP1 plus ZEB1 shRNA. **F**. RT-qPCR was used to detect the expression of ZEB1. **G** and **H** Transwell assay was performed to assess the migration and invasion of colon cancer cells. **p < 0.01; n = 3

Then we explored the interaction among circCSPP1, miR-431 and ZEB1. The potential complementary pairing sequence between miR-431 and the 3′-UTR of ZEB1 is presented in Fig. 5A. The results of luciferase reporter experiment indicated that miR-431 mimics significantly reduced the luciferase activity of cells carrying the WT ZEB1 3′-UTR (Fig. 5B). In addition, RT-qPCR and western blot analyses revealed that miR-431 mimics notably decreased ZEB1 expression at the mRNA and protein level in the cells; by contrast, miR-431 inhibitor increased ZEB1 expression (Fig. 5C and D). Moreover, RIP assay using the antibody against Ago2 confirmed the interaction between miR-431 and ZEB1 (Fig. 5E).

Subsequently, the interaction between circCSPP1 and ZEB1 was investigated using RT-qPCR. The results revealed that the overexpression of circCSPP1 promoted the expression of ZEB1. However, this phenomenon was completely reversed by transfection with miR-431 mimics or ZEB1 knockdown (Fig. 5F). Similarly, the promoting effects of circCSPP1 on cell invasion and migration were significantly inhibited by ZEB1 knockdown (Fig. 5G and H). Thus, these data illustrated miR-431 targeted ZEB1 in colon cancer cells.

### CircCSPP1 activates the cyclin D1/CDK4/Rb signaling pathway in colon cancer

**Fig. 6:**
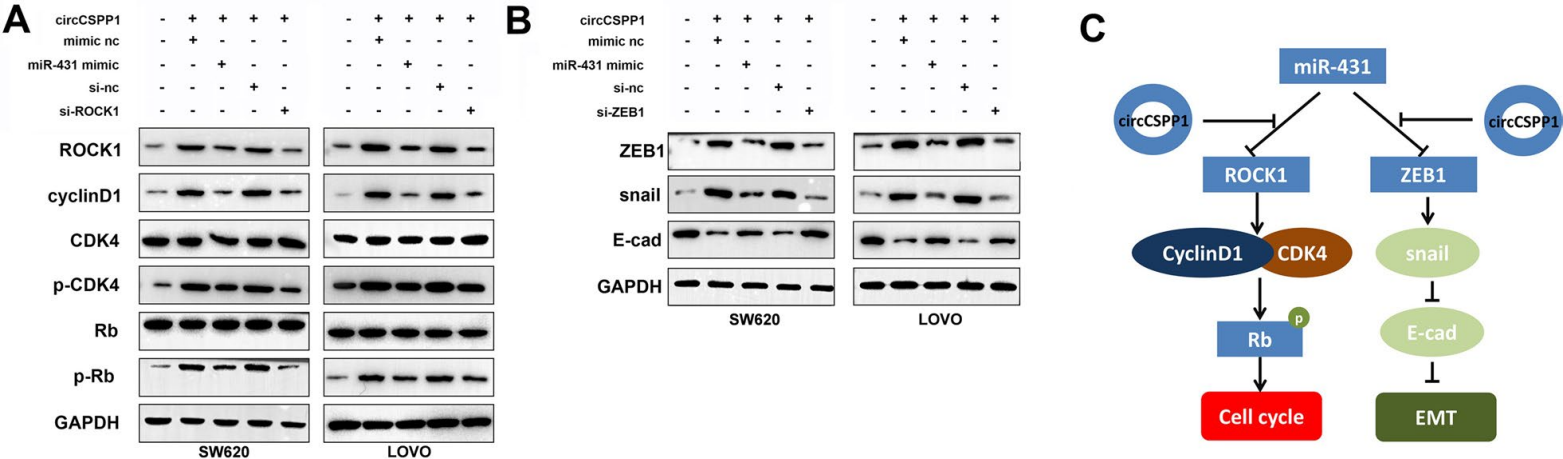
CircCSPP1 upregulates cyclin D1/CDK4/RB signaling pathway in colon cancer. Colon cancer cells were treated with pcDNA3.1-circCSPP1, pcDNA3.1-circCSPP1 plus miR-431 mimics or pcDNA3.1-circCSPP1 plus ROCK1 shRNA. **A**, **B** Western blot was used to detect the expressions of cell cycle-related proteins (cyclin D1, CDK4, Rb) and EMT related protein (Snail, E-cadherin) in each group. **C** The potential mechanism by which circCSPP1 regulated the progression of colon cancer was presented. N = 3

To further examine the role of the circCSPP1 in colon cancer, the levels of cell cycle-related proteins were detected using western blot analysis. The results indicated that the overexpression of circCSPP1 increased the expression of ROCK1, cyclin D1, p-CDK4 and p-Rb in the cells. However, these phenomena were reversed by transfection with miR-431 mimics or by ROCK1 knockdown (Fig. 6A). Moreover, it was found that circCSPP1 overexpression also increased the expression of ZEB1 and Snail, and downregulated the E-cadherin level. Similarly, the effects of circCSPP1 overexpression on these proteins were reversed by transfection with miR-431 mimics or by ZEB1 knockdown (Fig. 6B). In addition, circCSPP1 knockdown induced G1 arrest, while circCSPP1 overexpression promoted G1 arrest in colon cancer cells (Additional file 3: Fig. S3A and B). The potential mechanism through which circCSPP1 regulates the progression of colon cancer are presented in Fig. 6C. The schematic diagram illustrates that circCSPP1 promotes the progression of colon cancer by regulating the miR-431/ROCK1 and miR-431/ZEB1 pathways.

## Discussion

Increasing evidence has uncovered the critical role of circRNAs in the progression of human cancers, including colon cancer [19, 20]. Various studies have reported that circRNAs including circCCDC66 [27], circRNA_100859 [28] and circPPP1R12A [29] were upregulated in colon cancer and high expression was associated with a poor prognosis of the patients. In the present study, the dysregulated circRNAs were analyzed using the GEO database. A novel circRNA back-splicing 8–11 exons of the CSPP1 gene, termed circCSPP1, was found, which plays a tumor-promoting role in colon cancer.

Subsequently, the mechanisms of circCSPP1 regulating the progression of colon cancer were explored. The upregulated expression and the stability of circCSPP1 render it a potential biomarker and a diagnostic and therapeutic target in human cancers. CircCSPP1 was previous to be upregulated in liver cancer [30], prostate cancer [31], ovarian cancer [32]and glioma cancer [33]. In addition, circCSPP1 sponges different miRNAs including miR-520 h, miR-378 and miR-1182 to regulate tumor progression in these studies. The possible reason is that these miRNAs combine different specific 3ʹuntranslated regions of circCSPP1. These may indicate circCSPP1 is an active oncogenic factor. Similar to these reports, the present study found that circCSPP1 promoted the progression of colon cancer by sponging a new microRNA miR-431 and regulating the expression of ROCK1 and ZEB1.

ROCK1 and ROCK2 are Rho-GTPase effectors that control vital aspects of the actin cytoskeleton. The RhoA/ROCK pathway is activated in a variety of tumors and exerts a direct regulatory effect on the mobility of tumor cells [34–36]. Previous research has indicated that G1/S progression requires ROCK [37]. The role of ROCK1 in the regulation of the cell cycle may explain its effect on the proliferation of colon cancer cells. Our findings demonstrated that the knockdown of ROCK1 reversed the tumor-promoting effects of circCSPP1. The other function of ROCK1 is to induce the expression of cyclin-dependent kinase inhibitor (CDKI) p16, which prevents the CDK4/6-mediated phosphorylation of Rb proteins, thereby blocking E2F-dependent transcription [38]. The present study found that circCSPP1 promoted the expression of cyclin D1, p-CDK4 and p-Rb through the regulation of ROCK1 and miR-431. In addition, ZEB1 is well-known to be involved in the regulation of EMT in cancer cells [39]. In the present study, we found that circCSPP1 promoted EMT in colon cancer by modulating ZEB1.

In addition, recently, many studies have reported that circRNAs may encode proteins or peptides to participate in tumor progression [40], such as cricAXIN1 [41], circMAPK14 [42] and circCUX1 [43]. However, we did not study the encoding ability of circCSPP1. The issue is interesting, and it will be the focus of our further research.

In conclusion, the findings of the present study demonstrated that circCSPP1 was upregulated in colon cancer and functioned as an oncogene. In addition, circCSPP1 promoted the progression of colon cancer functions as a competing endogenous RNA by the regulating miR-431/ROCK1 and miR-431/ZEB1 pathways. The findings presented herein may provide novel insight into the pathogenesis of colon cancer. However, what factors regulate circCSPP1 to play a role in promoting cancer progression still need to be further studied. In addition, the potential translation function of circCSPP1 also needs further investigation.

## Supplementary Information

**Additional file 1**: Figure S1.CircCSPP1 targets miR-431 in colon cancer cells. **(A)** Luciferase reporter assay was performed to screen out the candidate miRNAs binding with circCSPP1. **(B)** The potential binding sites between miR-431 and circCSPP1. **(C)** Luciferase reporter experiment verified the binding relationship between circCSPP1 and miR-431. **(D)** The localization of miR-431 and circCSPP1 in colon cancer cells were detected with FISH experiment. **(E)** RIP experiment was carried out to confirm the interaction between circCSPP1 and miR-431. **(F)** RNA pull down was used to verify the interaction of circCSPP1 and miR-431. **p < 0.01; n = 3.

**Additional file 2**: Figure S2. MiR-431 targets ROCK1 in colon cancer cells. **(A)** The potential targets of miR-431 were predicted and the enrichment analysis was detected with RT-qPCR. **(B)** RT-qPCR was performed to analyze the expression of ROCK1 in colon cancer and adjacent normal tissues. **(C)** The potential binding sites between miR-431 and ROCK1 was presented. **(D)** Luciferase reporter experiment verified the binding relationship between miR-431 and ROCK1. **(E)** RIP experiment was carried out to confirm the interaction between miR-431 and ROCK1. **(F)** RNA pull down was used to verify the interaction of miR-431 and ROCK1. **p < 0.01; n = 3.

**Additional file 3**: Figure S3. CircCSPP1 regulates cell cycle in colon cancer. Colon cancer cells were treated with circCSPP1 shRNA2, shRNA-nc, pcDNA3.1-circCSPP1, or pcDNA3.1-control (scramble). **(A, B)** The cell cycle distribution was detected with PI staining method and flow cytometric analysis. **p < 0.01; n = 3.

**Additional file 4**: Figure S4. Cell transfection experiments. Colon cancer cells were treated with pcDNA3.1-circCSPP1, pcDNA3.1-control, shRNA-nc, ROCK1 shRNA, ZEB1 shRNA, miR-431 mimic or miR-431 inhibitor. **(A-E)** The gene expression of circCSPP1, ROCK1, ZEB1 and miR-431 in cells were detected with RT-qPCR. **p < 0.01; n = 3.

## Data Availability

The datasets used and/or analyzed during the current study are available from the corresponding author on reasonable request.
