# Supplementary figures and images for: Circular RNA circCSPP1 promotes the occurrence and development of colon cancer by sponging miR-431 and regulating ROCK1 and ZEB1
Source: J Transl Med. 2022 Jan 31;20:58. doi: 10.1186/s12967-022-03240-x (PMC8805259; doi:10.1186/s12967-022-03240-x)

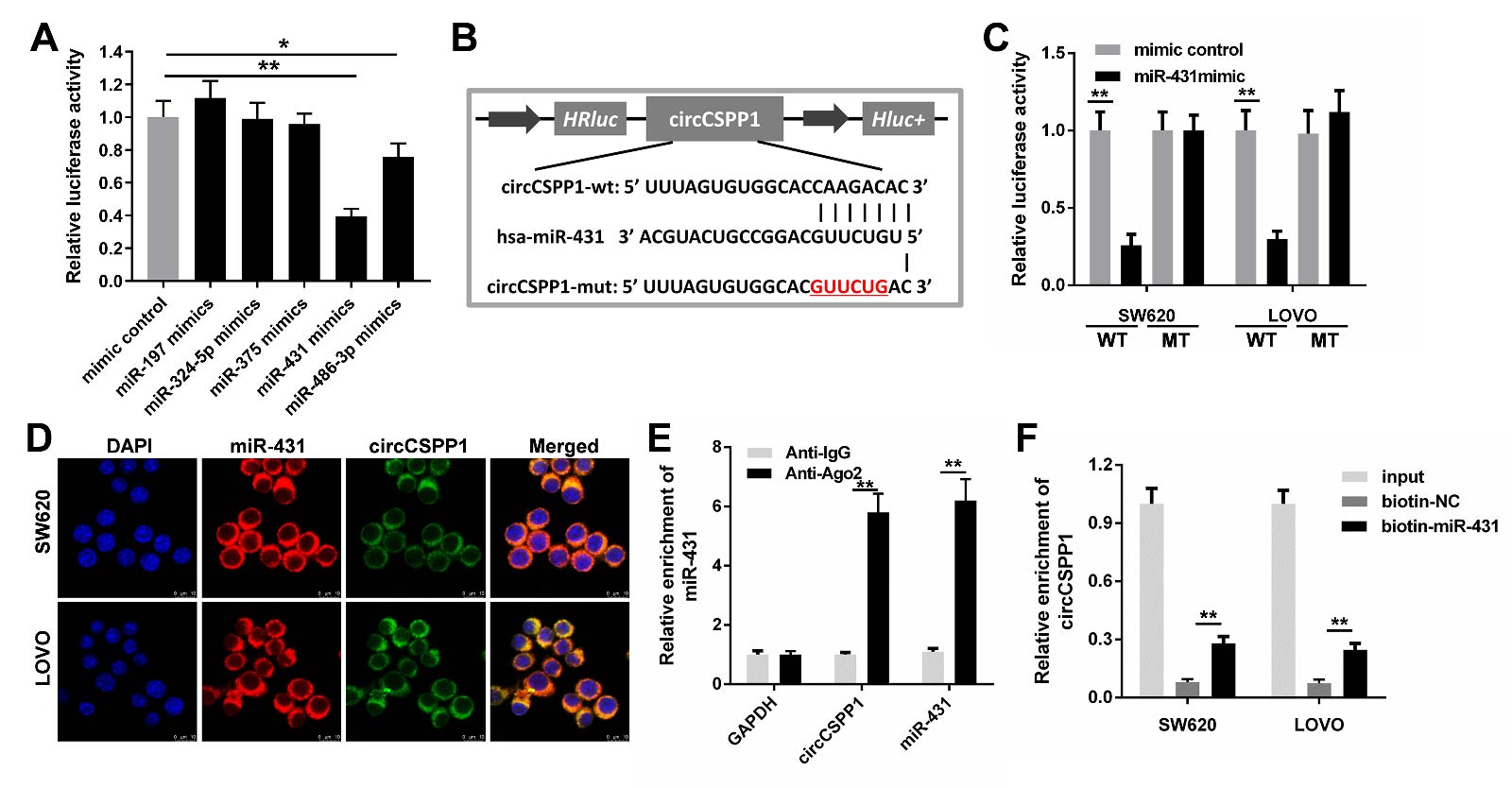

Supplement: Supplementary file 1 — Additional file 1: Figure S1.CircCSPP1 targets miR-431 in colon cancer cells. (A) Luciferase reporter assay was performed to screen out the candidate miRNAs binding with circCSPP1. (B) The potential binding sites between miR-431 and circCSPP1. (C) Luciferase reporter experiment verified the binding relationship between circCSPP1 and miR-431. (D) The localization of miR-431 and circCSPP1 in colon cancer cells were detected with FISH experiment. (E) RIP experiment was carried out to confirm the interaction between circCSPP1 and miR-431. (F) RNA pull down was used to verify the interaction of circCSPP1 and miR-431. **p < 0.01; n = 3. [file 12967_2022_3240_MOESM1_ESM.jpg]

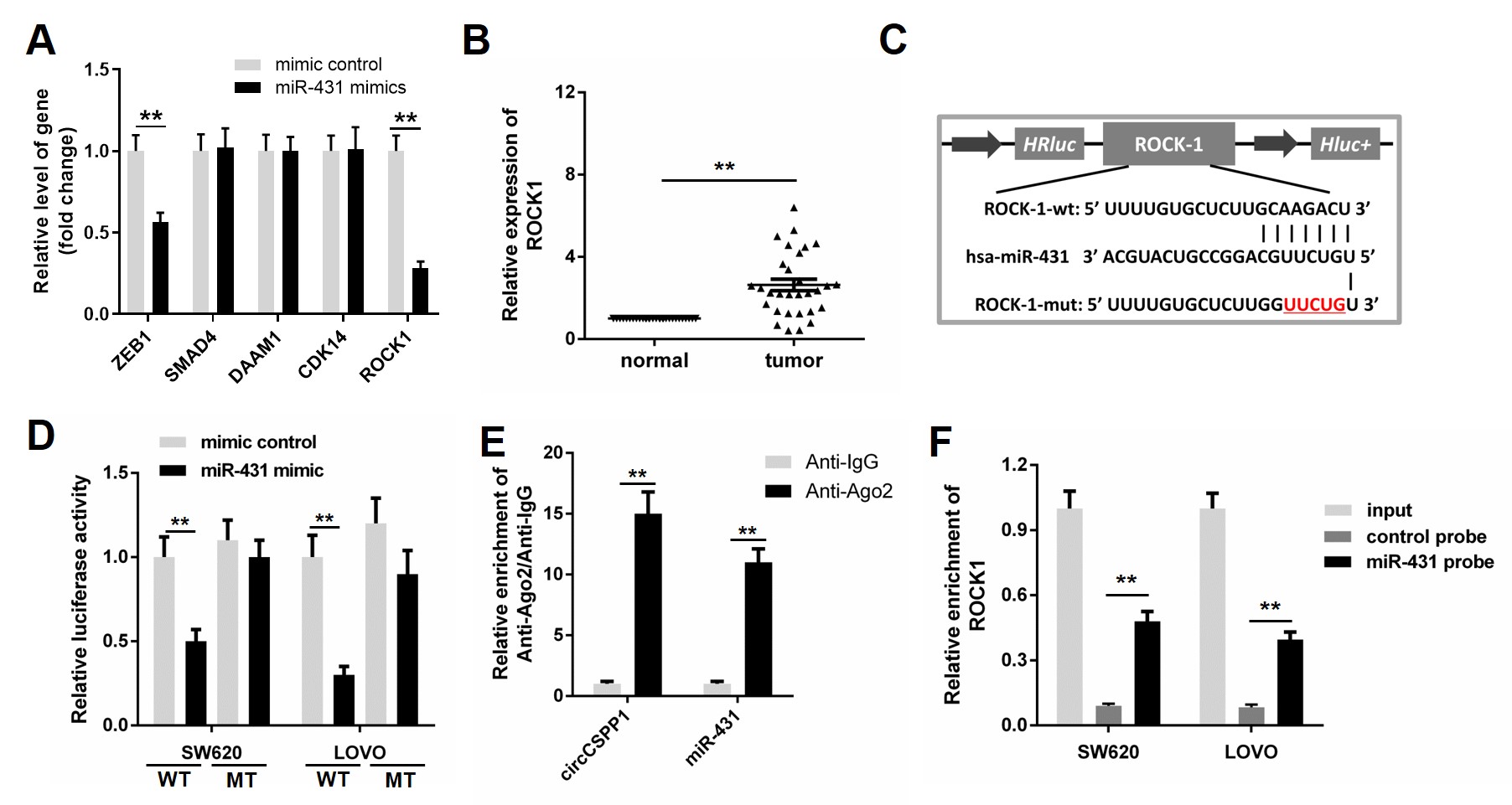

Supplement: Supplementary file 2 — Additional file 2: Figure S2. MiR-431 targets ROCK1 in colon cancer cells. (A) The potential targets of miR-431 were predicted and the enrichment analysis was detected with RT-qPCR. (B) RT-qPCR was performed to analyze the expression of ROCK1 in colon cancer and adjacent normal tissues. (C) The potential binding sites between miR-431 and ROCK1 was presented. (D) Luciferase reporter experiment verified the binding relationship between miR-431 and ROCK1. (E) RIP experiment was carried out to confirm the interaction between miR-431 and ROCK1. (F) RNA pull down was used to verify the interaction of miR-431 and ROCK1. **p < 0.01; n = 3. [file 12967_2022_3240_MOESM2_ESM.jpg]

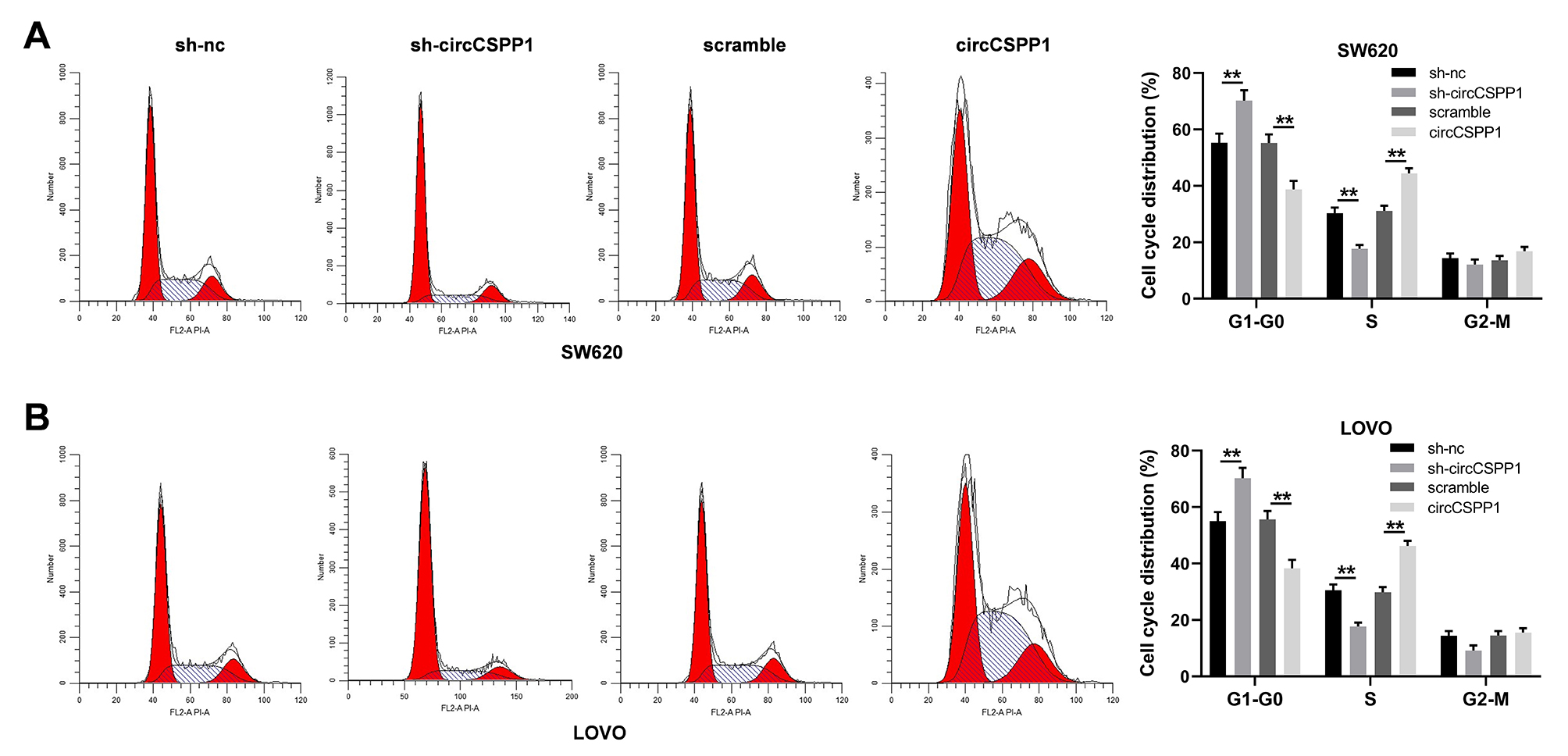

Supplement: Supplementary file 3 — Additional file 3: Figure S3. CircCSPP1 regulates cell cycle in colon cancer. Colon cancer cells were treated with circCSPP1 shRNA2, shRNA-nc, pcDNA3.1-circCSPP1, or pcDNA3.1-control (scramble). (A, B) The cell cycle distribution was detected with PI staining method and flow cytometric analysis. **p < 0.01; n = 3. [file 12967_2022_3240_MOESM3_ESM.jpg]

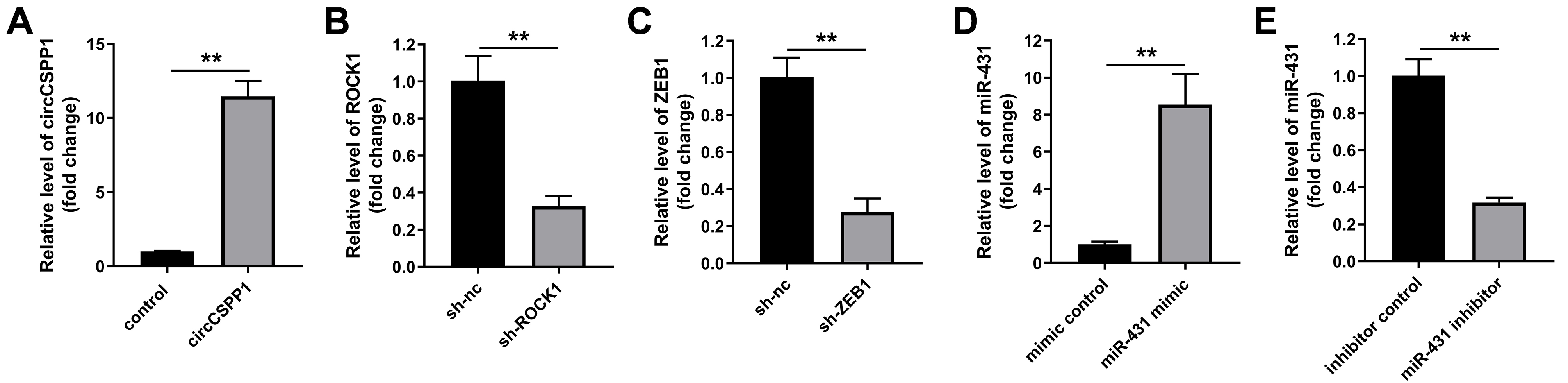

Supplement: Supplementary file 4 — Additional file 4: Figure S4. Cell transfection experiments. Colon cancer cells were treated with pcDNA3.1-circCSPP1, pcDNA3.1-control, shRNA-nc, ROCK1 shRNA, ZEB1 shRNA, miR-431 mimic or miR-431 inhibitor. (A-E) The gene expression of circCSPP1, ROCK1, ZEB1 and miR-431 in cells were detected with RT-qPCR. **p < 0.01; n = 3. [file 12967_2022_3240_MOESM4_ESM.jpg]
